# Half-Yearly Report of Cases in Midwifery, Which Have Occurred in the Northern District of the London and Southwark Midwifery Institution

**Published:** 1833-08

**Authors:** C. Waller

**Affiliations:** Consulting Accoucheur to the above Institution, and Lecturer on Midwifery at 93, Bartholomew close, near St. Bartholomew's Hospital.


### MIDWIFERY.

*Half-yearly Report of Cases in Midwifery, which have occurred in the Northern District of the London and Southwark Midwifery Institution.* By C. Waller, m.d., Consulting Accoucheur to the above Institution, and Lecturer on Midwifery at 93, Bartholomew close, near St. Bartholomew's Hospital.

| 1833. | Number of Women delivered. | Sex of Children. | | Born Alive. | Stillborn. | Presentation. |
|---|---|---|---|---|---|---|
| | | Males. | Females. | | | |
| January... | 47 | 20 | 27 | 45 | 2 | 45 Natural<br>2 Feet |
| February.. | 45* | 26 | 20 | 42 | 4 | 45 Natural<br>1 Placenta & Foot |
| March.... | 51 | 29 | 22 | 51 | 0 | 49 Natural<br>1 Breech<br>1 Foot |
| April ...... | 58† | 33 | 26 | 57 | 2 | 57 Natural<br>1 Face to Pubis<br>1 Breech |
| May ..... | 41 | 18 | 23 | 40 | 1 | 40 Natural<br>1 Breech |
| June ...... | 49 | 26 | 23 | 47 | 2 | Natural |
| Total...... | 291 | 152 | 141 | 282 | 11 | * Case of twins.<br>† Ditto. |

Several of the above, reported as Stillborn, were Premature Births.

It is satisfactory to be enabled to state that, during the past half-year, there has been a continuance of that freedom from danger amongst the patients of the charity mentioned in my last Report. One patient only has died; her disease was confirmed phthisis, which made very rapid progress after the delivery of the child, the fatal termination probably hastened in consequence of her labour being preceded by a considerable discharge of blood.

Two cases of peritonitis have occurred; one severe and extensive, the other of a more mild character, and of less extent. They were treated and cured by the usual means, viz. the removal of blood from the arm, followed by the application of leeches to the abdomen; after which a chamomile poultice enclosed within a flannel bag, and repeatedly changed, in order to keep up continued warmth and moisture, was directed to be applied, which not only acts as a fomentation, but also encourages the bleeding from the leech orifices, and, in consequence of its lightness, is more easily borne by patients than one made of bread or linseed-meal, the weight of which is such as to produce great pain when the peritoneum is extensively inflamed. The internal remedies were calomel and opium simply, beginning with a large dose, and repeating the medicine in small quantities, at short intervals, until the mouth was slightly affected. After ptyalism had been induced, the bowels were relieved with castor-oil, and the patients rapidly recovered. It is hardly necessary to state, that in these cases the abdominal inflammation was of the active character, the symptoms being widely different from those which take place in that truly terrific disease "puerperal fever," as it is called.

We have had several cases of slight hemorrhage, but the discharge was speedily arrested by the application of cold and friction.

The placenta presentation was very easily managed. The female was alarmed at a sudden discharge of blood, without pain, occurring at nearly the termination of gestation. On visiting her, there was no symptom of labour present; I therefore desired her to keep the recumbent position, and ordered her a little simple medicine. On calling the next day, I was surprised to find that she had got up, and gone out. In two or three days afterwards another summons was received; the bleeding having returned, and continued for some hours. On examination, the os uteri was discovered to be considerably dilated and very soft, the vagina relaxed, but there was no regular pain. On passing the finger within the uterus, a portion of the placenta could be distinctly felt, occupying about half the circumference of the os uteri, the membranes being readily distinguished at its anterior part. The female resided at some distance from my house, and I therefore thought it safer to finish the delivery, the parts being in so very favourable a condition. On introducing my hand, the first part of the child it came in contact with was the foot, so that the extraction was accomplished without the slightest difficulty. The uterus immediately contracted, and expelled the placenta; and from that period there was no hemorrhage.

Two severe cases of convulsions have happened; and in a third there were some very threatening head symptoms, which were relieved by bleeding. In both the other cases, the convulsions continued after the delivery of the child, but were eventually cured by bleeding from the arm, followed by leeches to the temples and free purging. Both females were in labour with their first child, and the infants were born dead: one presented the breech.

In one case the forceps were required. The female was in labour with her first child, and was twenty-eight years of age. The pelvis was well formed; the child, before the dilatation of the os uteri, had fallen into the cavity of the pelvis; there was a remarkable want of irritability in the uterus, there being little efficient pain. After she had been in labour for upwards of forty-eight hours, I deemed it better to finish the delivery; for, although the pulse was remarkably tranquil, there was pressure upon the bladder, impeding its functions. The head was extracted without difficulty. The patient required the use of the catheter for two days, but there was no other unpleasant symptom. It should be stated that abdominal friction was employed, and the Secale cornutum given; but they failed to arouse the dormant energies of the uterus.

One case is now under care which I fear will terminate fatally. The patient was seized with vomiting and spitting of blood towards the end of her pregnancy, over which remedies appeared to have no control, the stomach rejecting them, as well as every kind of food. She remained in this state upwards of a week, bringing up daily large quantities of blood. On inquiry, this female informed me she had been subject to these attacks in her two preceding labours, but that the symptoms subsided after the birth of the child, and did not again recur. Under these circumstances, I thought the hastening of the labour to be the plan most likely to afford relief, and accordingly punctured the membranes. The child, which was fullgrown and healthy, was born about sixteen hours after the evacuation of the waters; but the discharge of blood still continues, though in less quantities. The stomach irritability is also unsubdued, nearly the whole of her nourishment being rejected. Her countenance is blanched, and exhibits marks of distress, but her pulse is perfectly steady and low: this latter, indeed, is the only favourable symptom about her. The medicines employed have been saline effervescing draughts, calomel and opium, small doses of the neutral salts, hydrocyanic acid; and she is now trying tinct. ferri muriat. A blister has also been applied, but at present little or no benefit has been experienced.

*Bartholomew Close; July,* 1833.

